# Molecular and descriptive epidemiology of intestinal protozoan parasites of children and their pets in Cauca, Colombia: a cross-sectional study

**DOI:** 10.1186/s12879-019-3810-0

**Published:** 2019-02-26

**Authors:** Ximena Villamizar, Adriana Higuera, Giovanny Herrera, Luis Reinel Vasquez-A, Lorena Buitron, Lina Maria Muñoz, Fabiola E. Gonzalez-C, Myriam Consuelo Lopez, Julio Cesar Giraldo, Juan David Ramírez

**Affiliations:** 1 Grupo de Investigaciones Microbiológicas-UR (GIMUR), Programa de Biología, Facultad de Ciencias Naturales y Matemáticas, Universidad del Rosario, Bogotá, Colombia; 2 Centro de estudios en Microbiología y Parasitología (CEMPA), Departamento de Medicina Interna, Facultad de Ciencias de la Salud, Universidad del Cauca, Popayán, Colombia; 3 Departamento de Salud Pública, Facultad de Medicina, Universidad Nacional de Colombia, Bogotá, Colombia; 4 Programa de Biología, Universidad INCCA de Colombia, Bogotá, Colombia

**Keywords:** *Blastocystis*, *Giardia duodenalis*, *Cryptosporidium*, *Entamoeba histolytica/dispar/moshkovskii* complex, Zoonotic disease

## Abstract

**Background:**

Parasitic infections, particularly those caused by protozoa, represent a considerable public health problem in developing countries. *Blastocystis*, *Giardia duodenalis*, *Cryptosporidium* spp. and the *Entamoeba* complex (*Entamoeba histolytica, Entamoeba dispar* and *Entamoeba moshkovskii)* are the most common etiological causes of intestinal parasitic infections.

**Methods:**

We carried out a descriptive cross-sectional study in school-age children attending a daycare institution in commune eight of Popayán, Cauca (Southwest Colombia). A total of 266 fecal samples were collected (258 from children and eight from pets). *Blastocystis*, *G. duodenalis*, *Cryptosporidium* spp. and the *Entamoeba* complex were identified by microscopy, quantitative real-time PCR (qPCR) and conventional PCR. The concordance of qPCR and microscopy was assessed using the Kappa index. Molecular characterization was conducted to identify *Blastocystis* subtypes (18S), *G. duodenalis* assemblages (*tpi* and *gdh*) and *Cryptosporidium* species/subtypes (18S and GP60). Potential associations between intestinal parasitism and sociodemographic factors were examined using bivariate analyses.

**Results:**

A total of 258 fecal samples from children were analyzed by microscopy and 255 samples were analyzed by qPCR. The prevalence of *Blastocystis* was between 25.19% (microscopy) and 39.22% (qPCR), that of *G. duodenalis* was between 8.14% (microscopy) and 10.59% (qPCR), that of *Cryptosporidium* spp. was estimated at 9.8% (qPCR), and that of the *Entamoeba* complex was between 0.39% (conventional PCR) and 0.78% (microscopy). The concordance between microscopy and qPCR was very low. *Blastocystis* ST1 (alleles 4, 8, and 80), ST2 (alleles 11, 12, and 15), ST3 (alleles 31, 34, 36, 38,57, and 151), and ST4 (alleles 42 and 91), *G. duodenalis* assemblages AII, BIII, BIV and D, *C. parvum* subtype IIa and *C. hominis* subtype IbA9G3R2 were identified. The only identified member of the *Entamoeba* complex corresponded to *E. histolytica*. No statistically significant association was identified between parasitic infection and any sociodemographic variable.

**Conclusion:**

This study revealed the usefulness of molecular methods to depict the transmission dynamics of parasitic protozoa in southwest Colombia. The presence of some of these protozoa in domestic animals may be involved in their transmission.

## Background

Infections by Intestinal parasites occur worldwide, and their high prevalence rates represent a major public health problem. The major pathogens responsible for intestinal parasitic infections are protozoa. These microorganisms can cause significant morbidity in children as well as opportunistic infections in immunosuppressed patients [1]. Infection by intestinal parasites is generally associated with factors such as fecal contamination of soil and food, insufficient access to clean drinking water, lack of environmental sanitation, and vulnerable socioeconomic conditions. Given the socio-cultural features of developing countries, these regions tend to have the highest rates of infection by parasites [2]. Among the intestinal protozoa, *Blastocystis*, *Giardia duodenalis* (also known as *Giardia intestinalis* and *Giardia lamblia*), *Cryptosporidium* spp. and members of the *Entamoeba* complex (*Entamoeba histolytica, Entamoeba dispar and Entamoeba moshkovskii)* impose major burdens of diarrheal disease in children. The primary modes of protozoan parasite transmission are the fecal-oral route after direct or indirect contact with the infective forms (cysts/oocysts), human to human transmission, animal to human transmission, transmission by water, transmission through contaminated food, and airborne transmission (for *Cryptosporidium* spp. only) [3, 4].

*Blastocystis* are pleomorphic intestinal parasites commonly found in the gastrointestinal tracts of humans and both domestic and wild animals across the world [5]. Significant genetic diversity has been observed among the numerous *Blastocystis* isolates identified in humans and animals. *Blastocystis* can be grouped into subtypes with similar morphological characteristics: using the small subunit of ribosomal RNA (SSU rRNA), at least 17 subtypes (ST1 to ST17) and 151 different 18S alleles have been described [6–9]. ST1–ST8 and ST12 infect humans and animals (primates, pigs, cattle, rodents, and birds), ST9 infects only humans, and ST10, ST11, and ST13–ST17 have only been isolated from animals [10, 11]. In Colombia, the estimated prevalence of *Blastocystis* is 52.1% and studies have described the major circulating subtypes in human and animal populations (ST1–ST4 and ST6–ST8, of which ST1 and ST3 were found in humans and ST2 was found in both humans and dogs) [8, 11–13].

*G. duodenalis* is a single-celled flagellated parasite that infects the gastrointestinal tracts of humans and other mammals [14]. To date, eight genetic groups of *G. duodenalis* (assemblages A to H) have been identified [15]. Assemblages A (including AI and AII) and B (BIII and BIV) are responsible for most human infections and have also been identified in a wide range of mammals. The remaining assemblages show more restricted host ranges: assemblages C and D have been identified in canines, E in cattle, F in cats, G in rodents and H in seals and gulls [16]. In Colombia, the estimated prevalence of *G. duodenalis* in children is 15.4% and assemblages A and B have been detected with different frequencies depending on the population studied [13, 17–20].

*Cryptosporidium* spp. mainly infects the intestine and other extracellular spaces. Based on morphological, biological and molecular markers (SSU rRNA, HSP70, oocyst wall protein and the 60-kDa glycoprotein gp60, also known as gp40/15), at least 30 species and more than 70 genotypes have been identified. At least 20 species have been identified in humans and more than 90% of human infections are caused by *Cryptosporidium hominis* (anthroponotic) and *Cryptosporidium parvum* (zoonotic). Other species, including *Cryptosporidium meleagridis*, *Cryptosporidium canis*, *Cryptosporidium felis*, *Cryptosporidium ubiquitum*, and *Cryptosporidium cuniculus,* are less frequently detected in humans [21]. Ten subtypes of *C. hominis* (Ia–Ik) and 16 subtypes of *C. parvum* (IIa–IIp) have been described, with subtypes Ia, Ib, Id, and Ie of the former and subtypes IIa and IId of the latter having the highest prevalence worldwide [22]. In Colombia, the prevalence of this parasite is 0.5% and few studies have identified *C. parvum*, *C. hominis* or *C. viatorum* in humans [13].

The genus *Entamoeba* includes seven species: *E. histolytica*, *E. dispar*, *E. moshkovskii*, *Entamoeba bangladeshi*, *Entamoeba poleki*, *Entamoeba coli* and *Entamoeba hartmanni*. *E. histolytica*, *E. dispar* and *E. moshkovskii* are morphologically identical but genetically distinct; however, because the direct diagnostic methods currently in use do not permit their differentiation, they are typically reported as a complex. In humans the *Entamoeba* complex (*E. dispar*, *E. histolytica* and *E. moshkovskii*) is only differentiated by means of PCR. *E. dispar* is non-pathogenic while *E. histolytica* is pathogenic; the pathogenicity of *E. moshkovskii* is still controversial. In Colombia, all three species have been detected in asymptomatic children with a prevalence of approximately 15% [23].

Diagnosis of protozoan intestinal parasites is typically made using conventional methods such as microscopic examination of stool samples. This method is limited by its low specificity and sensitivity, which are related to the instability and rapid deterioration of some protozoan parasites outside the host [24]. Microscopic examination cannot distinguish between different species of *Cryposporidium*, between different assemblages of *Giardia*, between different subtypes of *Blastocystis* and between pathogenic and nonpathogenic species of *Entamoeba* [25]. For this reason, implementation of molecular techniques to diagnose circulating parasite species and subpopulations in vulnerable populations has been proposed to accurately measure parasite prevalence in endemic areas. Real-time PCR (qPCR) is an alternative technique that allows identification of parasitic DNA from preserved fecal matter and has a sensitivity between 80 and 100%, thus allowing more sensitive detection of parasitic infection than microscopy [24, 26, 27]. However, few studies in Colombia have addressed the prevalence of these parasites or attempted to compare prevalence estimates using different diagnostic techniques.

Therefore, the aim of the present study was the epidemiological and molecular characterization of intestinal protozoa (*Blastocystis, G. duodenalis, Cryptosporidium* spp. and members of the *Entamoeba* complex) in children and dogs living in the commune eight of Popayán, Cauca, southwest Colombia. A secondary goal was to conduct an overall comparison of the diagnostic performance of microscopy versus qPCR.

## Methods

### Study population

School-aged children (age 12–54 months) attending a daycare institution located in commune eight in Popayán, Cauca, southwest Colombia and 8 samples derived from dogs, as the only pet used in the study were recruited. A total of 266 fecal samples were collected (258 samples from children and eight from their pets). All samples were used for identification of intestinal protozoa by conventional and molecular methods. For samples that were positive using molecular methods, detailed molecular characterization was performed.

### Sociodemographic variables

At the time of providing informed consent, a structured survey was administered to collect information on the following variables: intestinal discomfort, socioeconomic stratum (In Colombia, the stratums are divided from 1 to 6 according to monthly income; stratums 1–2 are considered low-income, 3–4 middle-income and 5–6 high income), place of residence, age, sex, number of children in the house, monthly income, type of property, floor type, wall type, availability of public services, water quality, presence and number of pets, fecal elimination habits, hand washing habits and garbage storage/disposal procedures.

### Detection of intestinal parasites

#### Microscopy

Fecal samples were split into two. The first half of the sample was fixed with SAF solution (sodium acetate, acetic acid and formaldehyde) for identification of intestinal parasites by direct examination (microscopy) in saline solution containing Lugol’s iodine accompanied by the modified Ritichie-Frick concentration method and the Kato-Katz method as suggested by the World Health Organization (WHO) [28].

#### Real time PCR

DNA was extracted from the second half of the fecal sample using the Norgen Stool Extraction Kit, then The qPCRs were performed in 96 wells MicroAmp (Applied Biosystems), reactions in a total volume of 9 μL containing 3.5 μL of Taqman™ Mastermix (Roche), 1.0 μL of species-specific primers (10 μM) and primers of the internal amplification control (IAC) (10 μM), and 0.4 μL Taqman probes (5 μM) (*G. duodenalis, Blastocystis, Cryptosporidium*), 0.3 μL the water and 2.0 μL of DNA. The samples were processed by duplicate in an Applied Biosystems 7500 Fast equipment using default parameters of 40 cycles [13, 27, 29]. For the *Entamoeba* complex, conventional multiplex PCR was performed as previously reported [30]. We used DNA extracted from axenic cultures from *G. duodenalis, Blastocystis*, *E. histolytica, E. dispar, E. moshkovskii* and *C. hominis* as positive controls and fecal samples from patients from non-endemic regions that had previously tested negative for intestinal parasites by microscopy and qPCR as negative controls.

### Genotyping for identifying *G. duodenalis* assemblages, *Blastocystis* subtypes and alleles, and *Cryptosporidium* species and subtypes

Genotyping was conducted for samples that were positive by qPCR for *G. duodenalis*, *Blastocystis*, and *Cryptosporidium* spp.. For identification of *Giardia* assemblages, these samples were subjected to conventional PCR using primers specific for the following molecular markers: (i) *gdh* (glutamate dehydrogenase) using primers GDHeF (5′-TCAACGTYAAYCGYGGYTTCCGT-3′), GDHiF (5′-CAGTACAACTCYGCTCTCGG-3′) and GDHiR (5′-GTTRTCCTTGCACATCTCC-3′) as reported elsewhere [31], and (ii) *tpi* (triose phosphate Isomerase) using primers AL3543 (5′-AAATIATGCCTGCTCGTCG-3′), AL3546 (5′-CAAACCTTITCCGCAAACC-3′), AL3544 (5′-CCCTTCATCGGIGGTAACTT-3′), and AL3545 (5′-GTGGCCACCACICCCGTGCC-3′) as reported elsewhere [32]. For identification of *Blastocystis* subtypes and alleles, SSU rRNA was amplified using primers RD5 (5′-ATCTGGTTGATCCTGTCCAG-3′) and BhRDr (5′-GAGTGCCTTTTTAACAACAACG-3′) as previously described [33]. *Cryptosporidium* spp. were identified using direct sequencing of the SSU rRNA fragment using primers 18SF (5′-AGTGACAAGAAATAACA ATACAGG3′) and 18SRv (5′-CCTGCTTTAAGCACTCTAATTTTC-3′) [34]. Subtyping of *C. hominis* and *C. parvum* was based on sequence analysis of gp60 genes. Each specimen was analyzed by the relevant method at least twice. Subtype families for *C. hominis* and *C. parvum* were determined based on sequence differences in the nonrepeat region of the gene. Within each subtype family, subtypes differed from one another, mostly in the number of serine-coding trinucleotide repeats (TCA, TCG, or TCT microsatellite) located in the 5′ region of the gene. The previously-established nomenclature system was used to differentiate subtypes within each subtype family as reported elsewhere [10].

Both strands of PCR products were sequenced using the dideoxy-terminal method for all genetic markers herein employed in a 310 Genetic Analyzer (Applied Biosystems). Sequences were edited and aligned using MEGA 7.0 [35] and compared with reference sequences using BLAST. For *Blastocystis*, a database established for allele and subtype confirmation was queried (http://pubmlst.org/blastocystis/). In the case of *G. duodenalis*, a phylogenetic reconstruction was performed using maximum likelihood methods with 1,000 bootstrap replicates. Reference sequences contained in GenBank with the following accession numbers were included: AI (M84604), AII (AY178737), BIII (AF069059), BIV (AY178739), C (U60982), D (U60986), E (AY178741), E (AB182127), F (AB569384), G (AF069058), G (AY178745), H (GU176089). The phylogenetic tree was rooted with *Giardia ardeae* (AF069060). For *Cryptosporidium,* sequences were compared with species and subtype control sequences harbored at CDC, Atlanta and kindly provided by Dr. Lihua Xiao.

### Statistical analyses

Descriptive statistics were used to analyze data of interest. Variables were classified as categorical or continuous. Categorical variables were summarized by relative frequencies and their association with the presence of intestinal protozoa (*Blastocystis*, *G. duodenalis*, *Cryptosporidium* spp. and members of the *Entamoeba* complex) was assessed using chi-square tests. The 95% confidence intervals (CIs) were calculated for each of the associations. For continuous variables, the normality of the data was assessed using the Shapiro-Wilk test. Means and standard deviations were used to summarize normally-distributed variables, while medians and interquartile ranges were used for variables with non-normal distributions. The means and medians of each continuous variable were compared by t-tests or Mann Whitney U tests, depending on the fulfillment of the assumption of normality. All analyses were performed in STATA version 14.0 and values of *P* < 0.05 were considered statistically significant.

Concordance between microscopy and qPCR results was assessed by calculating the kappa index. A value of kappa close to one indicated that the results of both methods were concordant; and a value close to zero indicated that the methods were not concordant.

## Results

### Prevalence of intestinal parasitic infection

**Fig. 1:**
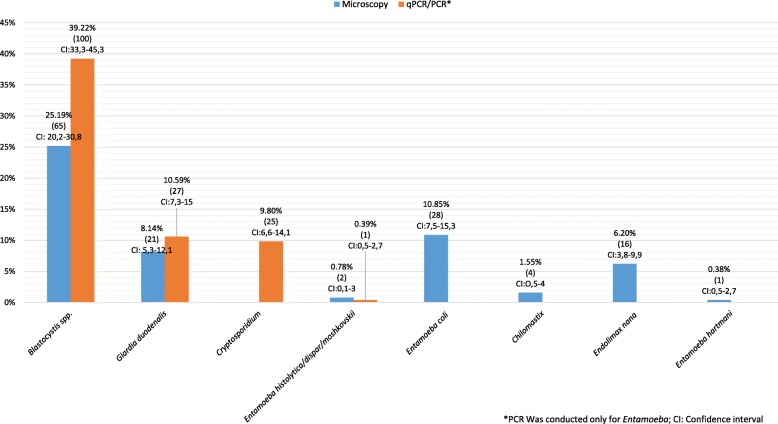
Frequency of intestinal parasites detected by microscopy, qPCR and PCR in the samples analyzed

A total of 258 human fecal samples were analyzed by microscopy and 255 were analyzed by PCR (only for *Blastocystis*, *G. duodenalis*, *Cryptosporidium* spp. and *Entamoeba* complex). Three samples were not able to be analyzed by qPCR due to the low amount of the fecal sample that only allowed microscopy examination. Prevalence estimates by microscopy were 25.19% (65/258) for *Blastocystis*, 8.14% (21/258) for *G. duodenalis*, 0.78% (2/258) for members of the *Entamoeba* complex, 10.85% (28/258) for *E. coli*, 1.55% (4/258) for *Chilomastix* spp., 6.20% (16/258) for *Endolimax nana* and 0.38% (1/258) for *Entamoeba hartmani* (Fig. 1). *Cryptosporidium* spp. could not be identified by microscopy due to logistical limitations. No helminths were detected across the samples. The presence of the most frequent intestinal protozoa parasites in Colombia was detected by qPCR. Prevalence estimates by qPCR were 39.22% (100/255) for *Blastocystis*, 10.59% (27/255) for *G. duodenalis*, 9.8% (25/255) for *Cryptosporidium* spp. and 0.9% (1/255) for *E. histolytica* (Fig. 1). Eight samples of pet feces were analyzed by qPCR and two samples showed evidence of infection by intestinal protozoa. *Blastocystis* was identified in one sample and in the other sample, *G. duodenalis* and *Cryptosporidium* spp. were detected; curiously, the owner of the *G. duodenalis* and *Cryptosporidium*-infected pet was not infected by either of these parasites, as was the case for the owner of the *Blastocystis*-infected pet.

### Evaluation of polyparasitism

**Fig. 2:**
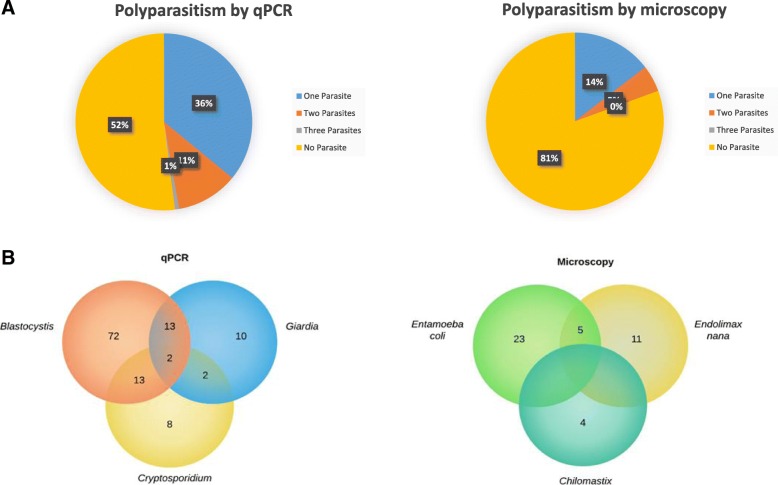
**a**. Percentage of stool samples positive for 0–3 parasites by qPCR and microscopy. **b**. Polyparasitism identified by qPCR of *Blastocystis*, *G. duodenalis*, and *Cryptosporidium* spp. and polyparasitism identified by microscopy for other parasites

We found that among the stool samples analyzed by qPCR (for detection of *Blastocystis*, *Giardia*, and *Cryptosporidium* spp.) and conventional PCR (for detection of the *Entamoeba* complex), approximately 36% of individuals were infected by a single parasite, 11% were infected by two parasites and 1% were infected by three parasites (Fig. 2a). Using microscopy, we found that *E. coli*, *Chilomastix* spp., *E. nana* and *E. hartmani* were involved in polyparasitism (Fig. 2a). Using qPCR, we found that *Blastocystis/G. duodenalis* and *Blastocystis/Cryptosporidium* (Fig. 2b) coinfections occurred more frequently than *G. duodenalis/Cryptosporidium* coinfections (*P* < 0.05). Members of the *Entamoeba* complex were not involved in polyparasitism.

### Comparison of the diagnostic performance of qPCR vs. microscopy

**Fig. 3:**
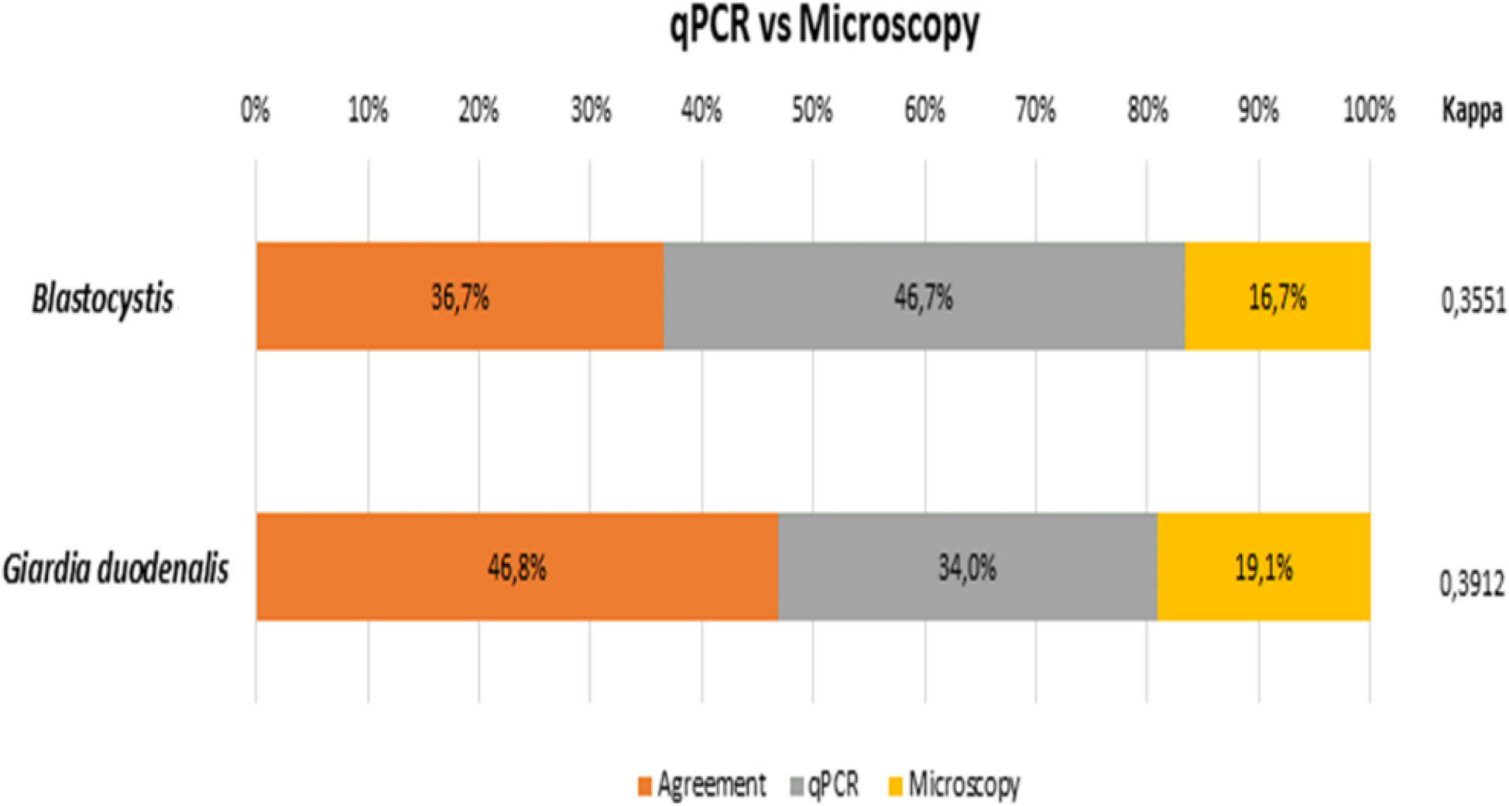
Analysis of concordance as shown by the kappa index between qPCR and microscopy measurements for identification of *Blastocystis* and *G. duodenalis*

We analyzed the concordance between the qPCR and microscopy results and determined that qPCR was significantly more sensitive than microscopy for identification of *Blastocystis* (71.7% versus 56.1%, P < 0.05) and *G. duodenalis* (90% vs. 83.6%, *P* < 0.05). For samples testing positive both by microscopy and qPCR, *Blastocystis* was identified in 36.7% (*n* = 44) of samples by both techniques, in 16.7% (*n* = 20) of samples only by microscopy and in 46.7% (*n* = 56) of samples only by qPCR. The overall concordance between the two techniques was low, with a kappa index of 0.3551 (Fig. 3). *G. duodenalis* was identified in 46.8% (*n* = 22) of samples by both techniques, in 19.1% (*n =* 9) of samples only by microscopy and in 34% (*n* = 16) of samples only by qPCR. The kappa index was 0.3912 (Fig. 3).

### *Blastocystis* subtypes, *Giardia duodenalis* assemblages and *Cryptosporidium* species

**Fig. 4:**
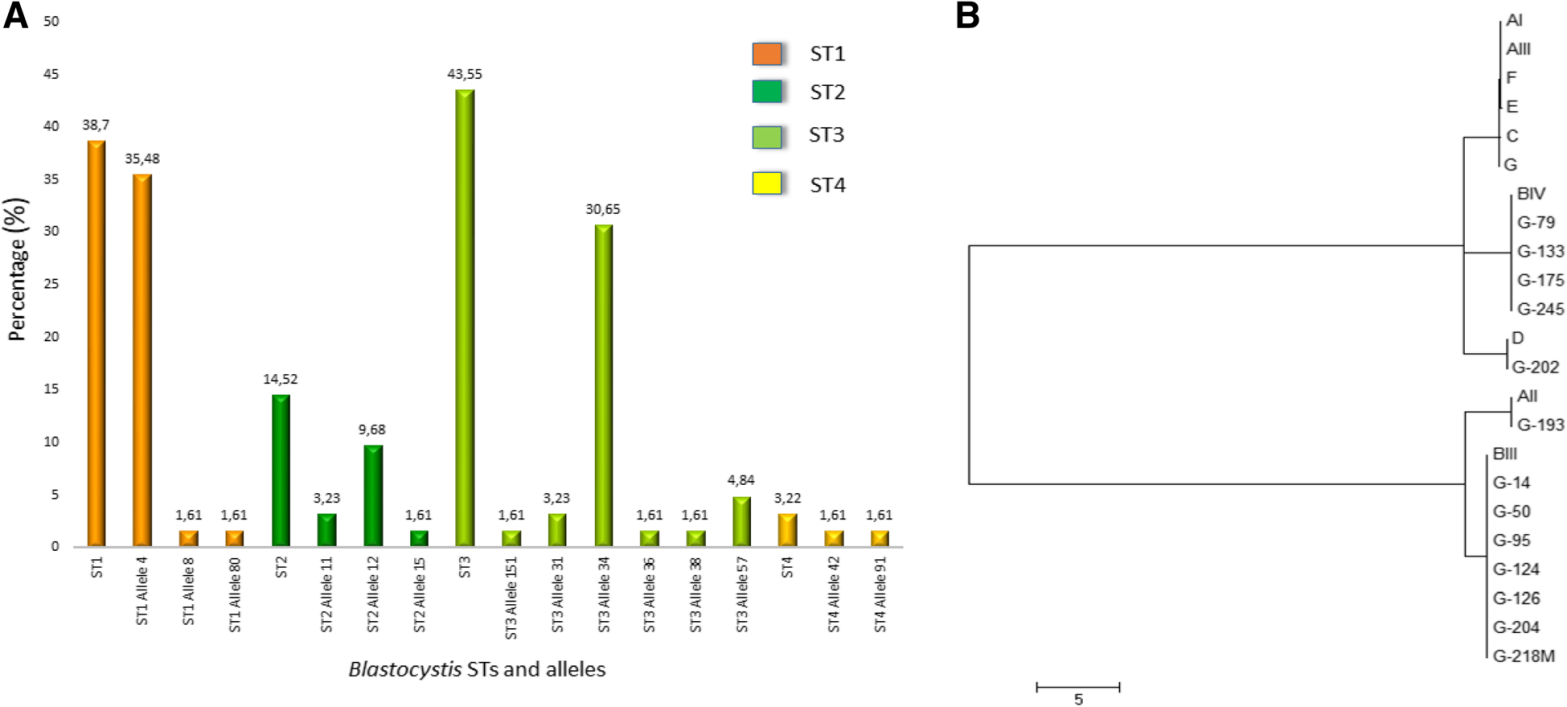
**a**. Frequency of *Blastocystis* subtypes and alleles. **b**. Phylogenetic relationships of *Giardia duodenalis* inferred using neighbor-joining methods applied to *gdh* nucleotide sequences

The prevalence rates of *Blastocystis* subtypes, *G. duodenalis* assemblages and *Cryptosporidium* spp. were assessed by genotyping samples that were positive by qPCR. A total of 62 *Blastocystis* samples that were qPCR-positive were successfully subtyped. The most common subtypes were ST1 (38.7%, *n =* 24), ST2 (14.52%, *n =* 9), ST3 (43.55%, *n =* 27) and ST4 (3.22%, *n =* 2). In addition, the different alleles associated with each subtype were identified. For ST1, alleles 4, 8, and 80 were identified; for ST2, alleles 11, 12, and 15 were identified; for ST3, alleles 151, 31, 34, 36, 38, and 57 were identified; and for ST4, alleles 42 and 91 were identified. ST1 allele 4 had the highest frequency, while the most frequent subtype was ST3 (Fig. 4a). The single pet fecal sample testing positive for *Blastocystis* was identified as ST1, allele 4 (Fig. 4a).

Of the 27 samples testing positive for *G. duodenalis* by qPCR, only 12 could be genotyped using the *gdh* gene as no amplification products for Sanger sequencing were obtained from the remaining 15 samples. In the case of the *tpi* gene, multiple bands were observed after electrophoresis and were subsequently unable to be sequenced. The *G. duodenalis* assemblages identified were AII (8.3%, *n* = 1), BIII (50%, *n* = 6), BIV (33.3%, *n* = 4) and D (8.3%, *n* = 1) (Fig. 4b). The single dog fecal sample testing positive for *G. duodenalis* was genotyped as BIII. We also observed coinfection between *Blastocystis* STs and *G. duodenalis* in six samples. Coinfection occurred between the *G. duodenalis* BIII assemblage and *Blastocystis* ST1, ST2, and ST3; between the *G. duodenalis* BIV assemblage and *Blastocystis* subtypes ST2 and ST3; and between *G. duodenalis* assemblage D and *Blastocystis* subtype ST1.

Although 25 samples were qPCR-positive for *Cryptosporidium* spp., only four samples could be genotyped for gp60 and three samples for SSU-RNA. In three samples, *C. parvum* subtype IIa was identified and in one sample, *C. hominis* subtype IbA9G3R2 was identified. In the case of the single positive dog fecal sample, *C. parvum* subtype IIa was identified.

### Association between sociodemographic variables and intestinal parasitism

Of the study population, similar proportions of participants were male (*n* = 134, 51.94%) and female (*n* = 124, 48.06%). The most common socioeconomic stratum of study participants was stratum one (80.62%), followed by stratum two (17.05%) and stratum three (2.33%). The vast majority (98.45%) of families had incomes lower than the minimum wage. However, 67.83% of families had their own homes, most with cement floors and brick walls, and 100% of the dwellings reported having an adequate system for sanitary elimination of excreta. Moreover, 99.61% of homes had access to water from treated aqueducts. The majority (88.76%) of children surveyed did not report gastrointestinal symptoms; only 6.2 and 3.49% reported diarrhea and abdominal pain, respectively. Characteristics of the study population are shown in Table 1. No statistically significant associations were identified between any variable and the presence of intestinal parasites.

**Table 1:** Sociodemographic variables studied under statistical analyzes

Variable	Category	*Blastocystis*			*Giardia duodenalis*			*Cryptosporidium*			*Entamoeba histolytica/dispar/moshkovskii*		
		Positive	Negative	*P* value	Positive	Negative	*P* Value	Positive	Negative	*P* Value	Positive	Negative	*P* Value
Sex	Female	28	96	0,352	9	115	0,618	12	111	0,98	0	124	0,172
	Male	37	97		12	122		13	119		2	132	
Stratum	1	49	159	0,305	17	191	0,746	22	184	0,543	2	206	0,785
	2	15	29		4	40		3	40		0	44	
	3	1	5		0	6		0	6		0	6	
Type of population	Urban	64	188	0,626	21	231	0,461	24	225	0,567	1	251	0,000
	Rural	1	5		0	6		1	5		1	5	
Children per housing	1	34	113	0,802	12	135	0,709	17	129	0,791	0	147	0,448
	2	26	65		9	82		7	84		2	89	
	3	3	10		3	13		1	11		0	13	
	4	1	4		0	5		0	4		0	5	
	5	1	1		0	2		0	2		0	2	
Pets	Yes	17	63	0,328	5	75	0,457	8	70	0,872	1	79	0,560
	No	48	130		16	162		17	160		1	177	
Water quality	Treated	65	192	0,561	21	236	0,766	25	229	0,741	2	255	0,929
	Non-treated	0	1		0	1		0	1		0	1	

*P*-value: < 0.05

## Discussion

Popayán, Cauca, a city in the southwest of Colombia, is blessed with significant forestry and mining resources. However, according to the last census, 22.2% of the population had unsatisfied basic needs, reflecting mainly a lack of housing and to a lesser extent, inadequate coverage of services such as education, health, drinking water and basic sanitation. These factors facilitate the development of multiple infections among the population, including intestinal parasites. Therefore, epidemiological studies such as the present one are imperative to understand the epidemiological and molecular features of the intestinal protozoa (*Blastocystis*, *G. duodenalis*, *Cryptosporidium* and *Entamoeba* spp.) affecting the infant population in developing countries.

The protozoa of interest were identified using both microscopy and qPCR. *Blastocystis* had the highest prevalence using both methods followed by *G. duodenalis* and *Cryptosporidium* spp. (detected only by qPCR) (Fig. 1). These findings are consistent with a national survey of parasitism, which showed that *Blastocystis*, *Giardia* and *Cryptosporidium* spp. were the most prevalent protozoa [36] and also with estimates from Latin America. Surprisingly, members of the *Entamoeba* complex, despite having the highest prevalence rates in the country, were detected at low frequency in our study regardless of the method of detection. When reviewing previous studies of intestinal parasites affecting the local population (Popayán, Cauca), we found no reports of the prevalence of members of the *Entamoeba* complex. Therefore, it is possible that the population studied here had a low prevalence of these parasites. However, the poor agreement between diagnostic methods (PCR and microscopy) for identification of *Entamoeba* complex members may be due to a high rate of false positives in microscopy because amoebic trophozoites can be easily confused with leukocytes (particularly macrophages that have phagocytosed red blood cells) and cysts of other amoebas [37].

Although the traditional method for diagnosis of gastrointestinal parasites is microscopy, it showed a low sensitivity for the identification of intestinal protozoa in our study compared with qPCR (Fig. 1). Likewise, comparison of these methods using the kappa index (for detection of *Blastocystis* and *G. duodenalis*.) showed a low concordance (Fig. 3). In the case of the *Entamoeba* complex, concordance could not be analyzed due to insufficient data, given the low prevalence of these parasites. Our concordance findings are similar to other studies, with a greater parasite prevalence detected by qPCR compared with microscopy [27, 38]. A study carried out by Sánchez et al. [13] among indigenous communities of the Amazon basin also showed low concordance between these methods. These results including evidence from Argentina and Ecuador support the use of molecular methods instead of microscopy for diagnosis of intestinal parasites [8] and for monitoring of patients post-treatment [39]. For logistical reasons, we did not attempt microscopic identification of *Cryptosporidium* spp. in the present study. It is important to note that this process is carried out using the Ziehl-Neelsen technique (modified acid-fast staining). This method has some disadvantages: it requires at least 50,000–500,000 oocysts per gram of fecal matter as well as significant operator expertise so as not to confuse the oocyst with other acid- and alcohol-resistant microorganisms of similar size such as *Cyclospora* or yeasts [40]. Further studies comparing microscopy with molecular methods should be conducted to establish the true prevalence of this protozoan because it has been underestimated in most developing countries. Because the immune systems of young children are not fully developed, they are more susceptible to infection and this could explain the high prevalence observed in our study. However, this parasite has been listed as one of the main etiological agents of diarrhea in children; this was demonstrated in a multicenter study conducted in Africa and Asia, where it was established as the second causative agent of diarrhea in children [41].

Polyparasitism was evaluated in the study population by qPCR and microscopy (Fig. 2). This polyparasitism may be caused by a variable immune response that may be influenced by nutritional status and repeated exposures to intestinal parasites [45]. Polyparasitism is the result of simultaneous infection with various helminths and intestinal protozoa, and is associated with ecological and environmental factors, different routes of infection and different exposures to the host [1]. Polyparasitism is very important for public health because it has a significant impact on general morbidity, nutritional status, immune reaction after treatment, and re-infection rates, causing an increase in the intensity of infection for most patients. Infection by multiple parasite species confers increased susceptibility to other infections [46], and qPCR allows greater sensitivity in the identification of polyparasitism, making it a useful tool for the evaluation of public health interventions [47]. In Colombia, studies of polyparasitism have been carried out in indigenous communities and found that both helminths and protozoa were involved [48].

We performed genotyping of the protozoa detected in our study and established that the circulating *Blastocystis* subtypes in the population were ST1, ST2, ST3, and ST4 (Fig. 4). The subtype with the highest prevalence was ST3. These results are consistent with two previous reports in Colombia, one study of nine localities in Colombia and another of indigenous communities of the Amazon region; both studies also identified the ST3 subtype as having the highest prevalence [12, 13]. Similarly, a study that included subtyped samples from several geographic regions around the world identified subtypes ST1 to ST9. Approximately 90% of the isolates belonged to ST1, ST2, ST3 and ST4 and ST3 caused most human infections worldwide [10]. We detected allele 4 of ST1 most frequently, as has been previously described in Colombia [8, 13]. Interestingly, we also detected ST4 allele 42, which has been previously reported in Colombia at low frequency, and allele 91, which has never been previously reported in the country. *Blastocystis* ST4 has a more restricted geographic distribution because of its more recent colonization of humans; thus, alleles of this subtype are rarely detected in humans, and the 91 allele has been reported only in a study of Danish patients [49, 50]. By contrast, *Blastocystis* ST3 has been commonly identified from humans and non-human primates, and given its relatively high specificity for these hosts, infections are assumed to be caused by human-to-human transmission [7].

*G. duodenalis* assemblages A, B and D were identified in our study, and sub-assemblies AII, BIII, and BIV had higher prevalence than the BIV assemblage. The distribution of assemblages was not geographically limited, given that several studies carried out inside and outside the country revealed widely different distributions [16]. In Colombia, two studies conducted in specific areas of the country (one of clinical samples collected between 1997 and 2001 in the departments of Amazonas, Boyacá and Bogotá, and another conducted in the Amazon region) revealed that the most prevalent assemblage was A [13, 17]. However, other studies conducted using stool samples in central and Caribbean Colombia demonstrated a higher prevalence of assemblage B, similar to our study [18–20]. Our study population, similar to a study conducted in the central region of Colombia [20], was largely asymptomatic. In both studies, there was no association between symptoms and presence of the *G. duodenalis* B assemblage; other studies of children in Australia and Brazil presented similar findings [51–54]. These findings are contradicted by other studies, which suggested an association between severe diarrhea and this assemblage [55, 56].

Surprisingly, in our population we identified *G. duodenalis* assemblage D (Fig. 4b). This assemblage has been mainly detected in canines; however, a study conducted in German travelers identified assemblage D in two human samples originating in South Asia [57]. None of the canine fecal samples included in our study showed evidence of infection by *G. duodenalis* assemblage D. Therefore, the infection in this patient was likely to be transient. To verify this hypothesis, we would need to re-collect a fecal sample to establish the course of the infection. It would be beneficial to carry out additional studies in canines and humans to clarify our knowledge of the transmission dynamics of uncommon assemblages in humans and to evaluate the traceability of infections caused by them. Regarding *Cryptosporidium* spp., *C. parvum* and *C. hominis* (subtypes IIa and IbA9G3R2, respectively) were identified. These findings are consistent with the literature, as *C. parvum* and *C. hominis* are responsible for 90% of human infections. Likewise, *C. parvum* subtypes IIa and Ib are prevalent worldwide, and subtype IIa is considered to be predominant in humans and other animals [21, 22, 58].

In this study, the following protozoa were identified in stool samples from dogs: *Blastocystis* subtype ST1 allele 4, *G. duodenalis* assemblage BIII and *C. parvum* IIa. When comparing these results with those obtained from children, we found that the child living with the *Blastocystis*-infected dog was also infected by the same *Blastocystis* subtype and allele. By contrast, the child living with the *Giardia*- and *Cryptosporidium*-infected dog was not infected with either of these two protozoa. However, each of these parasites has been previously identified in both animals and humans, indicating that in our study population zoonotic transmission may have given rise to some of the infections [42–44].

In Colombia, several studies of intestinal parasites have included the sociodemographic variables listed here (Table 1). These studies have reported a heterogeneous distribution of associations. A study conducted in Calarcá found a higher prevalence of intestinal parasitism in children who do not achieve growth and development controls and a significant association between infection with *Blastocystis* and non-deworming of pets [59]. Another study carried out in the indigenous reservation in Nasa, Cauca, did not find statistical associations between sociodemographic conditions and parasitism. However, the authors described factors such as: low education of parents and low availability of aqueduct and sewerage that may play a role [60]. Finally, a study carried out in preschoolers and schoolchildren in Cajamarca found an association between parasitism and level of education [61]. In our study, we did not find any statistically significant associations with demographic variables.

## Conclusions

The qPCR had better sensitivity for identification of *Blastocystis*, *G. duodenalis* and *Cryptosporidium* spp.*,* which were detected at considerable frequency in the study population, with a higher prevalence of *Blastocystis*. The subtypes and alleles distributed in the population were determined and we identified an uncommon allele reported in humans (allele 91 of *Blastocystis* ST4). *G. duodenalis* assemblage D was identified in human feces, although this assemblage is typically found in canines. This may have represented possible zoonotic transmission because we detected these protozoa in canine feces. Our findings provide information for control entities about the distribution and transmission dynamics of intestinal parasites, which may help in implementation of strategies to reduce their prevalence in children.

## Abbreviations

Blast: Basic Local Alignment Search Tool
Gdh: Glutamate dehydrogenase
Gp60: Glycoprotein 60
Mega: Molecular evolutionary genetics analysis
qPCR: Quantitative polymerase chain reaction
SSU rRNA: Small ribosomal ribonucleic acid subunit
ST: Sequence type
Tpi: Triose phosphate isomerase
